# AMP-RNNpro: a two-stage approach for identification of antimicrobials using probabilistic features

**DOI:** 10.1038/s41598-024-63461-6

**Published:** 2024-06-05

**Authors:** Md. Shazzad Hossain Shaon, Tasmin Karim, Md. Fahim Sultan, Md. Mamun Ali, Kawsar Ahmed, Md. Zahid Hasan, Ahmed Moustafa, Francis M. Bui, Fahad Ahmed Al-Zahrani

**Affiliations:** 1 Department of Computer Science and Engineering, Daffodil International University, Daffodil Smart City, Birulia, Dhaka, 1216 Bangladesh; 2 Health Informatics Research Lab, Department of Computer Science and Engineering, Daffodil International University, Daffodil Smart City, Birulia, Dhaka, 1216 Bangladesh; 3 Division of Biomedical Engineering, University of Saskatchewan, 57 Campus Drive, Saskatoon, SK S7N 5A9 Canada; 4 Department of Software Engineering, Daffodil International University, Daffodil Smart City (DSC), Birulia, Savar, Dhaka, 1216 Bangladesh; 5 Department of Electrical and Computer Engineering, University of Saskatchewan, 57 Campus Drive, Saskatoon, SK S7N 5A9 Canada; 6 Group of Bio-photomatiχ, Information and Communication Technology, Mawlana Bhashani Science and Technology University, Santosh, Tangail, 1902 Bangladesh; 7 Department of Human Anatomy and Physiology, The Faculty of Health Sciences, University of Johannesburg, Johannesburg, South Africa; 8 School of Psychology, Centre for Data Analytics, Bond University, Gold Coast, QLD Australia; 9 Department of Computer Engineering, Umm Al-Qura University, 24381 Mecca, Saudi Arabia

**Keywords:** Antimicrobials, Microorganisms, Bacteria, Machine learning, Pseudo-amino acid compositional, Deep learning, Antibiotic resistance, Biomedical engineering, Engineering

## Abstract

Antimicrobials are molecules that prevent the formation of microorganisms such as bacteria, viruses, fungi, and parasites. The necessity to detect antimicrobial peptides (AMPs) using machine learning and deep learning arises from the need for efficiency to accelerate the discovery of AMPs, and contribute to developing effective antimicrobial therapies, especially in the face of increasing antibiotic resistance. This study introduced AMP-RNNpro based on Recurrent Neural Network (RNN), an innovative model for detecting AMPs, which was designed with eight feature encoding methods that are selected according to four criteria: amino acid compositional, grouped amino acid compositional, autocorrelation, and pseudo-amino acid compositional to represent the protein sequences for efficient identification of AMPs. In our framework, two-stage predictions have been conducted. Initially, this study analyzed 33 models on these feature extractions. Then, we selected the best six models from these models using rigorous performance metrics. In the second stage, probabilistic features have been generated from the selected six models in each feature encoding and they are aggregated to be fed into our final meta-model called AMP-RNNpro. This study also introduced 20 features with SHAP, which are crucial in the drug development fields, where we discover AAC, ASDC, and CKSAAGP features are highly impactful for detection and drug discovery. Our proposed framework, AMP-RNNpro excels in the identification of novel Amps with 97.15% accuracy, 96.48% sensitivity, and 97.87% specificity. We built a user-friendly website for demonstrating the accurate prediction of AMPs based on the proposed approach which can be accessed at http://13.126.159.30/.

## Introduction

Antimicrobial peptides (AMPs) are crucial to the immune system, which develops a primordial defense mechanism. They exist in various eukaryotic organisms, including insects, greenery, and humans^1^. These peptides have virucidal, tumoricidal, fungicidal, and bactericidal properties^2^. AMPs have a short length (six to a hundred amino acid residues) and play a significant role in treating and preventing infectious diseases by focusing on harmful microorganisms^3^. AMPs have attained significant interest as a potential replacement of traditional medications such as chemotherapy, radiation therapy, fungus-based therapy, viral-based therapy, and so on^4,5^. In contrast to these traditional methods, AMPs are highly conducive to developing new methods with easier ways against these outdated techniques. Most of the researchers are still concerned about the detection of AMPs to discover the properties and create drugs based on each property, which are beneficial for the medical environment. Generally, AMPs are the walls of microbes and enter their cells to eliminate specific microorganisms. This approach guarantees the decimation of microbes and minimizes the likelihood of developing drug resistance^6^. The identification of AMPs using traditional biochemical and biological methods is time-consuming and expensive. Therefore, researchers have constructed various datasets such as the Antimicrobial Peptide Database (APD), APD3, Data Repository of Antimicrobial Peptides (DRAMP), ADAM, LAMP and so on from AMPs and made predictions using computational methods^7–13^.

In 2017, Meher et al. proposed a sequence-based statistical predictor with the compliance of Chou’s 5-step rule to discover the most crucial features associated with the functional activity of AMPs and they named the proposed predictor iAMPpred^14^. However, they used the correlation coefficient between amino acids and order-related rational data. Their approach could be a linear relationship, which may not produce satisfactory results for complex biological interactions. In 2018, Veltri et al. applied a Deep Neural Network (DNN) approach to detect AMPs. The authors used the Bag of Words (BoW) method to obtain numerical values from peptides^15^. In 2019, Su et al. proposed a Multi-Scale Deep Neural Network (MS DNN). At first, they used a Long Short-Term Memory (LSTM) approach with different layers. However, their approach provided insufficient results; therefore, they fused the MS DNN with the traditional model to find AMPs^16^. In the same year, another method was proposed by Wei et al.^17^. The authors used Graph Attention Networks (GAT) to detect peptide sequences using Skip-Gram and Word2Vec to create numerical numbers^17^. However, they did not consider the information derived from each amino acid's specific location or position within a sequence. In 2021, Xiao et al. constructed a two-level predictor called the iAMP-CA2L structure using a Convolutional Neural Network (CNN) and Support Vector Machine (SVM) to classify AMPs and instead quasi-classify them into 10 relevant AMP subcategories^18^. In 2022, Li et al. proposed a deep learning model, named AMPlify, based on Bi-directional Long Short-term Memory (Bi-LSTM) to predict the AMPs^19^. According to the study, their proposed model suffered from notable shortcomings, namely a lower sensitivity which is a greater gap between sensitivity and specificity. In another study, Dee et al. built an LMpred predictor based on pre-trained language and deep learning methods to classify AMPs^20^. However, the authors obtained insufficient performances with this model, and there is still room for improvements to detect the AMPs. In 2023, Yen et al. constructed a sAMPpred-GAT model based on the graph attention approach^21^. However, the model was performed with insufficient performances with complex strategies, and as such there are still opportunities to improve the accuracy with lower complexity. Xu et al. proposed an iAMPCN framework based on deep-learning methods, where the authors employed a two-stage procedure to distinguish AMPs and their functionalities^22^. In the same year, another study proposed by Lee et al. developed a Bidirectional Encoder Representations from Transformers (BERT)-based framework called AMP-BERT^23^. In another study, Söylemez et al. designed an AMP-GSM framework to detect AMPs based on grouping, scoring, and modeling stages^24^. Panwar et al. developed a GEU-AMP50 framework based on Artificial Neural Network (ANN) and multiple machine-learning algorithms to detect the AMPs^25^. In another study in the same year, Yang et al. constructed an AMPFinder model based on a deep-learning approach^26^.

Therefore, according to the above survey of recent studies, there is still a significant potential for improving the accuracy and robustness of AMP localization with the availability of a wide range of computational approaches in this field. In this study, we applied a novel approach called AMP-RNNpro to detect AMPs. The advancement of our approach includes the following steps:

1. This study applied CD-HIT to reduce the redundancy of the combined dataset containing 10,600 sequences, which are extracted with eight feature encoding methods.
2. We applied 33 models in each feature extraction and selected six best models with their overall performance.
3. To benefit from the individual strengths of each model, we generated the probabilistic features from these six models and integrated them to form the input layer as 48D of our meta-model.
4. This study introduced SHAP-based features, which are essential for the detection the AMPs and targeting therapeutic departments.

Our model, AMP-RNNpro, significantly outperforms other state-of-the-art methods. We have developed an efficient prediction framework based on our proposed model; the model can be accessed at http://13.126.159.30/.

## Methods

### Workflow of the study

**Figure 1:**
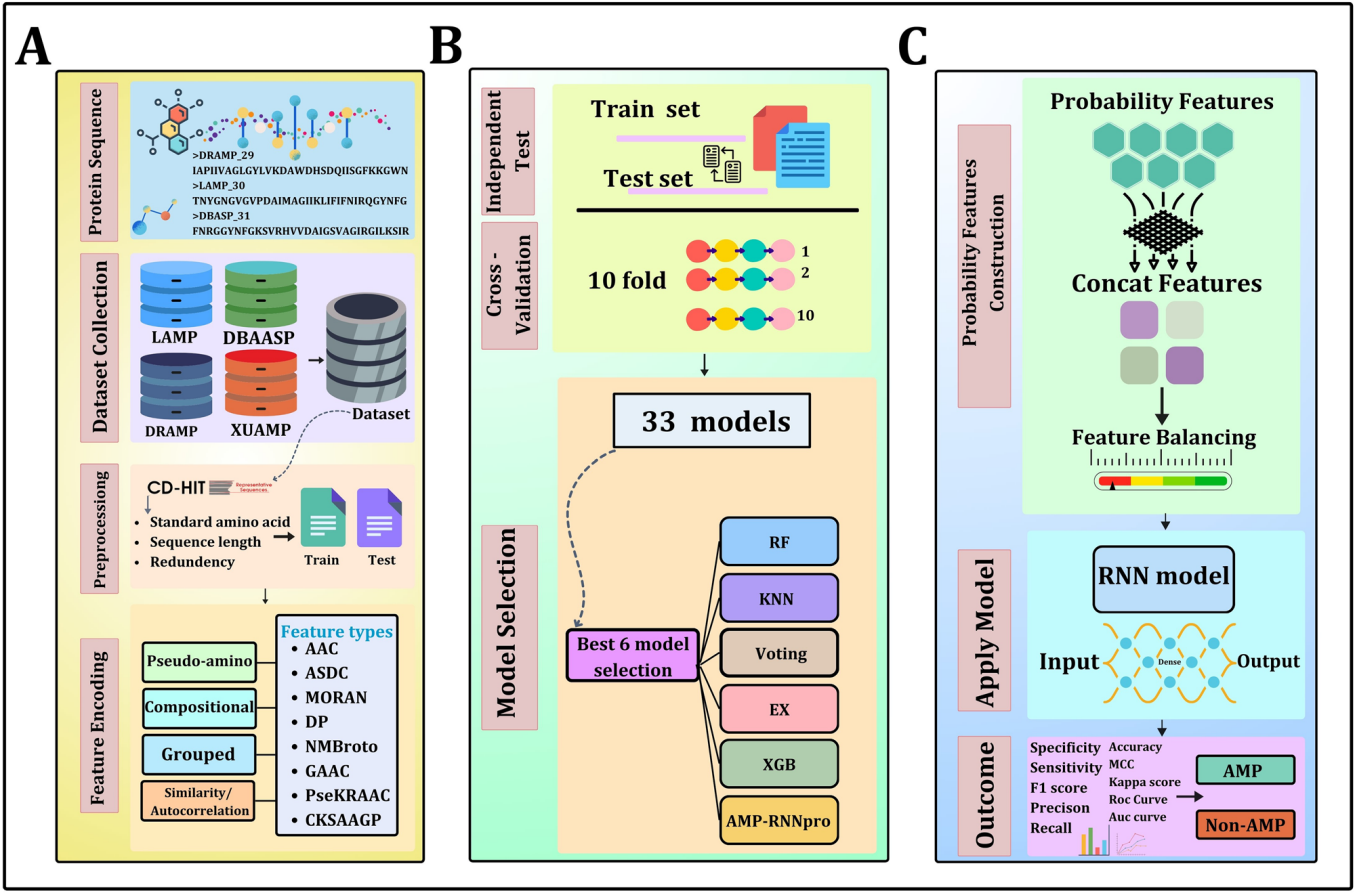
Overview of AMP-RNNpro method (**A)** Dataset collection, preprocessing phase, and feature encoding. **(B)** Applying these feature encodings on independent test and cross-validation methods with 33 individual models, then selecting six best models from 33 models. **(C)** Probability feature construction, deployment of RNN as the final predictor, and illustration of outcomes.

This study introduces a novel approach to identifying AMPs based on a comparatively larger dataset constructed and acquired through a comprehensive literature review. Our procedural methodology is depicted in Fig. 1. We have applied the CD-HIT to reduce the redundancy of the sequences to obtain a more furnished dataset. Eight feature extraction methods have been employed on the finalized dataset. We trained and tested machine-learning approaches by utilizing 33 methods on each of the eight feature encodings. The performance of the models was rigorously tested using independent tests and tenfold cross-validation strategies. To construct the secondary dataset, we selected six models based on their overall performances: K-nearest Neighbor (KNN), Random Forest (RF), Extreme Gradient Boosting Classifier (XGB), Extra-trees Classifier (EX), and two meta-classifiers, Voting Classifier (Voting), and a Recurrent neural network (RNN) based approach called AMP-RNNpro. All the models and relevant parametric variables were derived using Scikit-learn, a freely available data-mining library for Python^27,28^. Based on the eight feature encoding methods, we generated probabilistic values from the selected models, yielding 48 dimensional (48D) features fed into the final predictor. In the secondary dataset (48D probabilistic values), there have been more positive values than negative ones. Consequently, we used a balancing strategy called the Synthetic Minority Oversampling Technique (SMOTE) for the negative class^29^. Afterward, we fed the balanced dataset into six models, and according to the comparison results of these models, the AMP-RNNpro model has emerged as our meta-model of choice, given that it has taken 48D features as input and provided the most efficient outcomes. Finally, our methodology incorporates SHapely Additive exPlanation (SHAP) techniques to illustrate the top 20 features^30^, which significantly contribute to our model's performance.

### Dataset description

We collected four datasets for this study. Initially, we collected XUAMP data as our first dataset from Xu et al.^3^. The authors constructed their dataset by merging samples from several repositories such as the DRAMP^11^, DRAMP 2.0^31^, LAMP^13^, YADAMP^32^, etc. They selected 3072 samples with a sequence homology of less than 40%. As we constructed numerous datasets, we collected the second dataset from Yan et al.^21^. The authors created the DBAASP non-redundant independent test dataset by curating positive classes from DBAASPV3^33^ and negative classes from the UniProt databases^34^. In the DBAASP dataset, the authors obtained 356 samples, with the positive samples reducing the redundancy by 90% homology and the negative dataset by 40% homology. Accordingly, we gathered another dataset LAMP^13^ and DRAMP^11^. As mentioned, the XUAMP dataset has already been used to build their databases with a 40% threshold. In the current study, we merged all the datasets and applied the Cluster Database at High Identity with Tolerance (CD-HIT)^35^ with an 80% threshold and 5-word size. This procedure was conducted to reduce redundancy and increase efficiency in both the training and test datasets. This comprehensive selection of datasets guarantees a thorough and accurate evaluation of the capabilities of the proposed technique under various circumstances. Table 1 lists the statistical information of the datasets.

**Table 1:** Datasets and statistical information.

Dataset	Category	Positive	Negative	Total
Before CD-HIT	Train dataset	3536	3536	12,520
	Test dataset	3122	2326	
After CD-HIT	Train dataset	2865	3348	10,600
	Test dataset	2389	1998	

**Figure 2:**
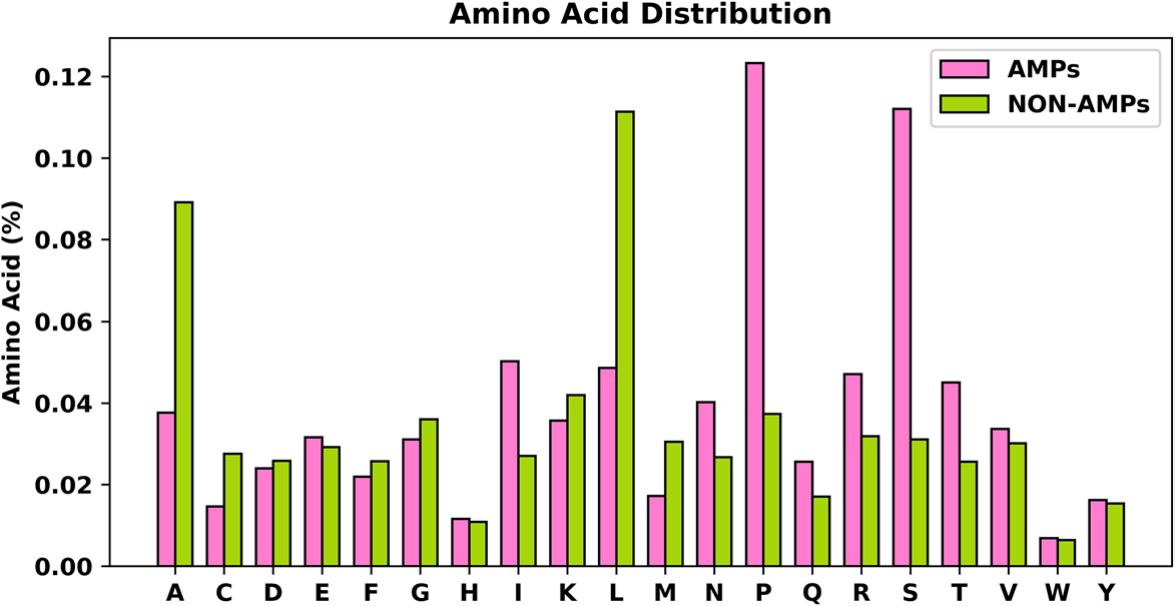
Compositional distribution of amino acid for both positive and negative sequences of the merged dataset.

Generally, the length of the sequences was not greater than 100 or less than 10. However, sequences with non-conventional amino acids, such as "B, J, O, U, X, Z” are rarely found^15^. These sequences were excluded while performing our study. The peptide protein sequences obtained were focused on "A, C, D, E, F, G, H, I, K, L, M, N, P, Q, R, S, T, W, Y" and filtered for further analysis. Figure 2 illustrates the amino acid distribution of the final datasets.

Figure 2 exhibits the compositional distribution of 20 amino acids in percentage for both positive and negative cases. The corresponding letters in the Fig. 2 indicates all the amino acids. There are 9 (nine) non-polar amino acids such as alanine (A), phenylalanine (F), glycine (G), isoleucine (I), leucine (L), methionine (M), proline (P), valine (V), and tryptophan (W). There are 6 polar, uncharged amino acids such as serine (S), cysteine (C), asparagine (N), glutamine (Q), threonine (T), and tyrosine (Y). Two amino acids are present in the acidic amino acid group. They are glutamic acid (E) and aspartic acid (D). Accordingly, lysine (K), arginine (R), and histidine (H), are essential amino acids^36^. In this study, we observed significant differences in the amino acid composition of active antimicrobial peptides (AMPs) and their inactive antimicrobial peptides (non-AMPs), as demonstrated by the bar graph analysis. We observed that in the positive AMPs, the non-polar amino acid proline (P) and the polar amino acid group serin (S) were enriched by more than 100%. For non-AMPs, the non-polar groups alanine (A) and leucine (L) constituted more than 80% of the total amino acids. In addition, tryptophan (W) appeared at lower levels in AMPs and non-AMPs.

### Feature encoding

Feature encoding methods play a vital role in the biological fields to prepare the datasets for machine learning and deep learning algorithms. Therefore, we employed eight feature encoding methods from four different feature encoding groups. The applied feature encoding groups and feature encoding methods are Amino Acid Composition (AAC), Adaptive Skip Dinucleotide Composition (ASDC), PseAAC of Distance-Pairs and Reduced Alphabet (DP) from the amino acid compositional group: Grouped Amino Acid Composition (GAAC) and The Composition of k-spaced Amino Acid Pairs (CKSAAGP) from Grouped amino acid compositional group, Moran (Moran) and Normalized Moreau-Broto (NMBroto) from the Autocorrelation-based feature encoding group: and Pseudo *K*-tuple reduced amino acid composition (PseKRAAC) from Pseudo-amino acid compositional-based feature group^37,38^.

### [I] Amino acid compositional features

#### AAC

The AAC calculates the normalized quantities of each amino acid sequence. It provides an overview of the proportion of each peptide^39^. The mathematical formula is as follows:

1 $$AAC\left(k\right)=\frac{{N}_{k}}{N}, \left(k \epsilon A,C,D\dots \dots .W,Y\right)$$

where $k$ denotes certain kinds of amino acids, ${N}_{k}$ is the length of the sequences, and $N$ is the total number of amino acids. In this study, we used 20D of the AAC features.

#### ASDC

ASDC is an adapted version of the dipeptide composition that generates a comprehensive descriptive process that considers all pertinent data between neighboring residues and intervening residues^39^. The feature vector of the ASDC can be defined as

2 $$A\left({f}_{i}\right)={\frac{\sum_{G=1}^{T-1}{O}_{i}^{G} }{{\sum }_{i=1}^{400}.{\sum }_{G=1}^{T-1}{O}_{i}^{G} } ,{(f}_{i} \epsilon f}_{1},{f}_{2},{f}_{3},\dots {f}_{400})$$

where $T-1$ is the interference amino acids, ${f}_{i}$ is the frequency of peptides, which is ≤ T − 1 intervening of amino acids, this study used 400D of the ASDC features.

#### DP

Another feature-encoding method is DP. This is based on the frequencies of k-spaced amino acid pairs, and the composition of the protein sequence and distance pairs used in PseAAC, which indicates pairs of amino acids that are detached by a certain quantity of residues. The Reduced Alphabet Scheme uses amino acids like clusters to reduce the dimensionality of the feature vector^40^. This formula is expressed as follows:

3 $$DP\left(i,j\right)=\frac{1}{N-\left(n+1\right)}{\sum }_{k=1}^{N-\left(n+1\right)}.{w}_{k}{,}_{i}{w}_{k+n,j}$$

where $DP(i,j)$ is the number of the distance pair of peptides, $N$ is the length of the sequence, $n$ is the distance between two peptides, and $w$ is the weight of the ${i}$ th residue at ${k}$ th sequences. In this study, the 20D DP features were used.

### [II] Grouped amino acid compositional features

#### GAAC

GAAC features are split into five groups: aliphatic groups with GAVLMI (6 amino acids), aromatic groups with FYW (3 amino acids), positively charged groups with KHR (3 amino acids), negatively charged groups with DE (2 amino acids), and group 5 with uncharged groups with STCPNQ (6 amino acids)^39,41, 42^. The mathematical formula can be specified as

4 $$G\left(k\right)=\frac{{N}_{k}}{N}, \left(G \epsilon { G}_{1},{G}_{2},{G}_{3},{G}_{4},{G}_{5}\right)$$

5 $$G\left(k\right)=\sum {N}_{k}, k \epsilon G$$

where $k$ is the acid type, $G$ is the group number, $N$ is the total number of acids, and $G(k)$ is the groups number of peptides. In this study, we used 5D of the GAAC features.

#### CKSAAGP

CKSAAGP considers amino acid combinations detached according to any k residues, giving a more adaptable way to identify local sequence trends in protein sequences. It includes evaluating the presence of amino acid groupings within a specified distance and potentially finding significant morphological and functional patterns^43^. The formula can be defined as:

6 $$C=\left(\frac{N\left[g1g1\right]}{T-\left(i+1\right)},\frac{N\left[g1g2\right]}{T-\left(i+1\right)},\dots \frac{N\left[g5,g5\right]}{T-\left(i+1\right)}\right)$$

where *T* means the length of peptides, *N* is the total number of acids, and *g1, g2,… g5* is the group of amino acids. 100D CKSAAGP-based features have been used in this study.

### [III] Autocorrelation features

#### MORAN

This is a mathematical correlation-based feature^44^ used to evaluate the closely related nearby measurements in a spatial data collection^45^. In this study, we used 16D features of the MORAN feature. The formula can be stated as:

7 $$M=\frac{N\sum_{k=1}^{N}.\sum_{j=1}^{N}{\beta }_{kj} \left({a}_{k}-\overline{a }\right).({a}_{j}-\overline{a })}{T{\sum }_{k=1}^{N}. ({a}_{k}-\overline{a }{)}^{2}}$$

where $T$ is the total quantity of the position at ${\beta }_{kj}$, *N* is the current number of coordinates, $\overline{a }$ is the normalized value of ${a}_{k, }{a}_{j}$ parameter, and ${\beta }_{kj}$ is the dimension of the coordinates.

#### NMBroto

This is similar to the MORAN feature. However, their differences lie in function, normalization, and calculation as NMBroto is calculated using the frequencies of k-spaced amino acid pairs and the amino acid composition of the protein sequence^46^. NMBroto can be defined as:

8 $${N}_{lagi}=\frac{1}{t-lag}[{\sum }_{k=1}^{t-lag}.\left({A}_{ik}\times {A}_{k}+la{g}_{i}\right)], k=\text{1,2},3\dots t-lag$$

where $k$ denotes the position of peptides. $A,t,lag$ denote the length of the residues and the distance between the peptides. This study used the 16D feature of the NMBroto.

### [IV] Pseudo-amino acid compositional features

#### PseKRAAC

This is an extension of the Pseudo Amino Acid Composition PseAAC. This feature has 16 types of clustering methods; in this study, we used type 7 features, also called multiple clusters, with 4 clustering methods^47^. The formula can be defined as:

9 $$P={f}_{i}={\sum }_{j=1}^{{20}^{n}}\frac{{f}_{i,j}}{{w}_{j}(N-n+1)}$$

where $w$ is the weight of the ${j}$ th position, $n$ is the length of the tuple, $N$ is the length of the sequence, and *fi* is the frequency in the ${i}$ th residue.

### Our proposed model construction

RNN is one of the most popular deep learning models used in various fields to detect the classes accurately^48^. RNNs can handle sequential or natural language processing (NLP) data. At each step, RNN possesses the internal layer of the input and the hidden state from the previous phase. This invisible state enables the recollection of the network and allows it to verify correlations in sequential input^49^. We selected this process for the optimal outcome to detect the AMPs, as RNNs are mainly used for the time series data, though could be utilized for sequence data, thus rendering them appropriate for jobs requiring sequential information. RNNs are intended to identify relationships and patterns in sequential data. FASTA patterns might vary in dimension, and RNNs can handle sequences of varied lengths despite requiring set input weights. This adaptability is significant in genetics and bioinformatics, where sequences might change in length.

**Figure 3:**
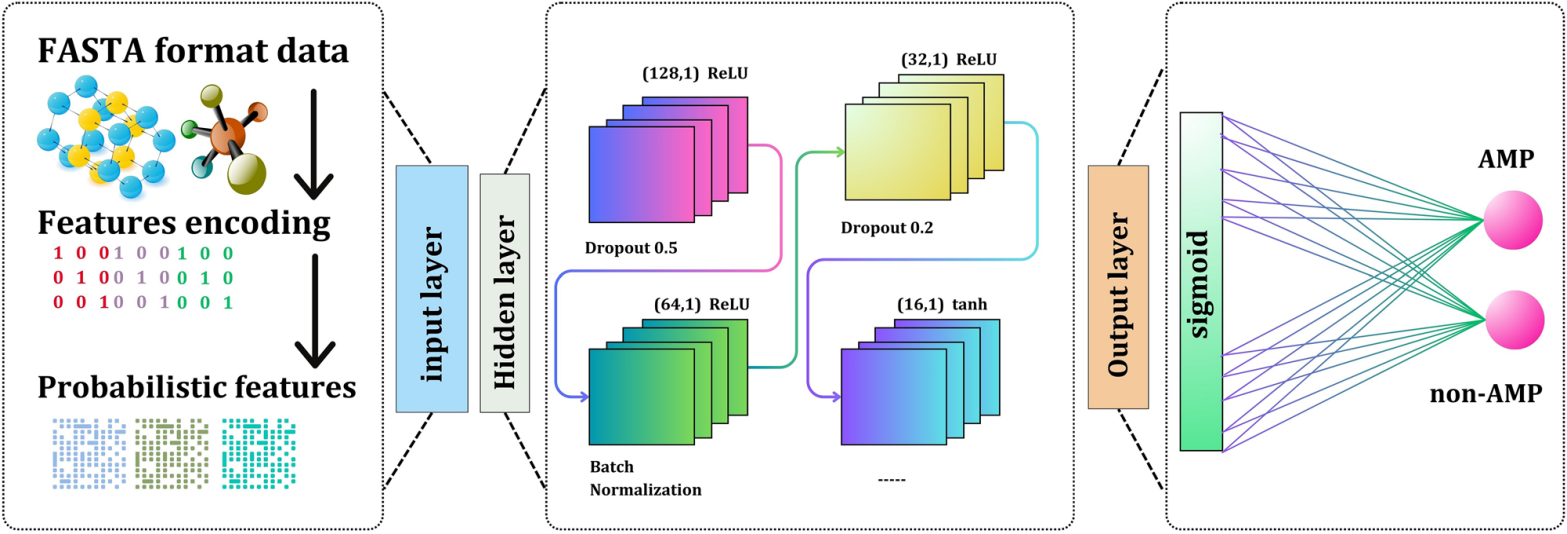
AMP-RNNpro framework’s structure overview.

We have constructed our meta-model “AMP-RNNpro” as shown in Fig. 3, that is optimized with six layers—an input layer, four hidden layers, and a dense layer. Accordingly, fifty epochs, three activation functions, and various filter sizes have been used in the independent test. The filter sizes connected with these layers are 128, 64, 32, and 16. We adopted the ReLU activation function in the first three layers, and in the fourth layer, we used the tanh function to handle the complexity. We added dropouts of 0.5, 0.2, 0.2, and 0.2 to reduce over-fitting. Finally, a dense layer contains a single neuron with a sigmoid activation function, producing binary numbers 0 and 1. A test result indicates an AMP if it is greater than 0.5; otherwise, it suggests a non-AMP. This study used Adam Optimizer to adjust the model's internal parameters. Notably, the Keras library, a popular tool for developing and upgrading neural networks, was used to compute our model^50^. The RNN structure, sigmoid function, tanh, and ReLU formulas are specified as:

10 $$Rnn= \alpha \left( {W}_{hh}{j}_{t-1}+{W}_{xh}{x}_{t}+{b}_{h }\right)$$

11 $$Sigmoid= {S}_{a}=\frac{1}{1+{e}^{-a}}$$

12 $$ReLU={R}_{u}= m\left(0,u\right) \left(\because u= u>0\right)$$

13 $$tanh(t)= {H}_{t}=\frac{{e}^{t}-{e}^{-t}}{{e}^{t}+{e}^{-t}}$$

where ${W}_{hh}$ is the matrix weight of the recurrent connections*,* ${W}_{xh}$ is the input connection weight, ${b}_{h}$ denotes the bias vector, *j* is the current state, ${j}_{t-1}$ is the previous state, and $\alpha$ is the activation function, ${R}_{u}$ denotes the ReLU*,* $m$ is the maximum, where it returns the maximum value between 0 and $u$, and $u$ is the input. ${S}_{a}$ denotes the sigmoid function, where $e$ presents the exponential function and the output range (0,1). ${H}_{t}$ is the tanh function; this function range is (−1, 1), ${e}^{t}-{e}^{-t}$ denotes the hyperbolic sine, and ${e}^{t}+{e}^{-t}$ denotes the hyperbolic cosine function.

### Machine-learning models

This study used 33 models, where we applied some traditional models and some meta-models using stacking classifiers, voting classifiers, along with simple RNN model. We investigated several combinations of voting and stacking based models. All the models are demonstrated in the supplementary file (S1). Among them we selected two meta-classifiers, Voting and AMP-RNNpro, additionally, four distinct classification methods, including K-nearest Neighbor (KNN), Random Forest (RF), Extreme Gradient Boosting Classifier (XGB), and Extra-trees Classifier (EX) based on their performance, and we have employed several hyper-parameters to obtain a better outcome. These models are further described in the following.

KNN is one of the most widely used classification techniques. In general, KNN analyzes most classes between the data points "K" in the feature area or the nearest data^51^. We set the K as 100 neighbors to account for the 100 nearest neighbors in the data sets. To obtain the distance between the data points, we applied the Manhattan technique. We used the weights parameter as distance for deciding whether closet neighbors had a more substantial impact on the prediction with their weights. Accordingly, we used the "kd tree” algorithm for the final dimension results.

Another classification technique, RF, predicts the result using the voting stage to generate many decision-making structures during the training phase^52^. In this study, the RF model is configured with "sqrt" as the feature dimension, where the number of features boosts the model's robustness and prevents over-fitting. The node splitting threshold was set at "entropy," predictability for repeatable outcomes was set at a random state value of "100," and the prediction method employed was an ensemble of "100" decision trees (DT) to extract feature information from the feature-dimensional selection.

The XGB model combines a highly streamlined operation with the potential of gradient-boosting method, where this method of tree construction and each subsequent tree address the mistakes made by its predecessors to produce an accurate result^53^. This study used an estimator of “100” for the number of boosts and a learning rate of “0.1"; a subsample of “1.0” denotes all training samples applied in each round. The regularization parameter is “30” for preventing underfitting or overfitting.

In the EX classifier builds the trees using random split techniques and provides the result by combining methods^54^. Where, EX provided the most effective results by the averaging method’s. This study used a “100” estimator for the classification.

Another popular ensemble approach in machine learning is the Voting classifier, where included have included the estimated probabilities across multiple baseline models such as KNN, RF, XGB, DT, and EX, which are subsequently employed as input data and used voting soft parameter to deliver the final classification results.

### Performance evaluation metrics

We measured the model’s effectiveness using the following metrics: Accuracy, Sensitivity (Sn), Specificity (Sp), Matthews Correlation Coefficient (MCC), Kappa Score (K), F1 Score (FS), and Precision (PR). These indicators allowed for a thorough quantitative assessment of the model’s performance. In this context, TP, TN, FP, FN denotes respectively true positive, true negative, false positive, and false negative^55–57^. The corresponding mathematical formulae are as follows.

14 $$Accuracy=\frac{\left(TP+TN\right)}{\left(TP+TN+FP+FN\right)}$$

15 $$PR=\frac{TP}{\left(TP+FP\right)}$$

16 $$Sn=\frac{TP}{\left(TP+FN\right)}$$

17 $$FS=\frac{2*\left(PR* RE\right)}{\left(\left(PR+RE\right)\right)}$$

18 $$MCC =\frac{TP*TN-FP*FN}{\sqrt{TP+FP*\left(TP+FN\right)*\left(TN+FP\right)}}$$

19 $$K=\frac{2*\left(TP*TN-FP*FN\right)}{\left(TP+FP\right)*\left(FP+TN\right)+\left(TP+FN\right)*\left(FN+TN\right)}$$

20 $$Sp=\frac{TN}{\left(TN+FP\right)}$$

## Experimental results

In this study, we have used several performance evaluation metrics as mentioned in the previous section to justify the performances of the developed models. We compared the performances of several machine learning models with our proposed model AMP-RNNpro. All the results have been compared and analyzed in this section, highlighting the performances of the proposed model.

### Performances of machine learning models

Table 2 demonstrates the independent test method for providing a better outcome than the cross-validation. In the supplementary file, we have added the cross-validations and other independent test performances accordingly.

**Table 2:** Performance of machine learning classifiers and AMP-RNNpro on feature encoding methods.

Descriptor	Classifier	Accuracy (%)	MCC (%)	K (%)	PR (%)	FS (%)	Sn (%)	Sp (%)	P-value
AAC	EX	**95.19**	90.58	90.36	95.19	95.19	98.50	92.42	< 0.01
	RF	94.67	89.50	89.31	94.67	94.67	97.75	92.09	< 0.01
	KNN	92.02	84.80	84.10	92.02	92.02	98.30	86.77	< 0.01
	XGB	75.86	53.09	52.15	75.86	75.86	83.88	69.15	< 0.01
	Voting	86.62	74.18	73.38	86.62	86.62	93.39	80.95	< 0.01
	AMP-RNNpro	95.17	90.43	90.30	95.17	95.17	97.60	93.14	< 0.01
ASDC	EX	95.42	90.95	90.81	95.42	95.42	98.00	93.26	< 0.01
	RF	95.24	90.58	90.44	95.24	95.24	97.80	93.09	< 0.01
	KNN	91.54	83.98	83.16	91.54	91.54	98.40	85.81	< 0.01
	XGB	88.97	78.23	77.93	88.97	88.97	92.69	85.85	< 0.01
	Voting	93.07	86.47	86.14	93.07	93.07	97.20	89.62	< 0.01
	AMP-RNNpro	**95.58**	91.22	91.12	95.58	95.58	97.65	93.85	< 0.01
CKSAA- GP	EX	93.18	86.56	86.35	93.18	93.18	96.40	90.50	< 0.01
	RF	93.37	86.97	86.72	93.37	93.37	96.90	90.41	< 0.01
	KNN	89.86	80.83	79.84	89.86	89.86	97.50	83.47	< 0.01
	XGB	84.43	69.64	68.99	84.43	84.43	90.39	79.45	< 0.01
	Voting	90.49	81.61	81.04	90.49	90.49	96.05	85.85	< 0.01
	AMP-RNNpro	**93.62**	87.31	87.19	93.62	93.62	95.80	91.80	< 0.01
DP	EX	**95.19**	90.58	90.36	95.19	95.19	98.50	92.42	< 0.01
	RF	94.83	89.77	89.62	94.83	94.83	97.50	92.59	< 0.01
	KNN	92.02	84.80	84.10	92.02	92.02	98.30	86.77	< 0.01
	XGB	75.86	53.09	52.15	75.86	75.86	83.88	69.15	< 0.01
	Voting	86.62	74.18	73.38	86.62	86.62	93.39	80.95	< 0.01
	AMP-RNNpro	95.17	90.44	90.31	95.17	95.17	97.70	93.05	> 0.01
GAAC	EX	89.72	79.88	79.46	89.72	89.72	94.29	85.89	< 0.01
	RF	89.58	79.44	79.16	89.58	89.58	93.19	86.56	< 0.01
	KNN	89.88	80.40	79.81	89.88	89.88	95.50	85.18	< 0.01
	XGB	60.25	24.68	22.67	60.25	60.25	78.63	44.87	< 0.01
	Voting	88.65	78.19	77.40	88.65	88.65	95.35	83.05	< 0.01
	AMP-RNNpro	**89.99**	80.67	80.05	89.99	89.99	95.85	85.10	< 0.01
MORAN	EX	90.18	81.13	80.43	90.18	90.18	96.45	84.93	< 0.01
	RF	90.13	80.96	80.32	90.13	90.13	96.05	85.18	< 0.01
	KNN	85.16	73.55	70.80	85.16	85.16	98.80	73.75	< 0.01
	XGB	70.62	44.45	42.36	70.62	70.62	84.68	58.85	< 0.01
	Voting	86.62	75.68	73.59	86.62	86.62	98.30	76.85	< 0.01
	AMP-RNNpro	**90.18**	81.02	80.41	90.18	90.18	95.95	85.35	< 0.01
NMBroto	EX	90.15	81.09	80.38	90.15	90.15	96.45	84.89	< 0.01
	RF	**90.18**	81.12	80.42	90.18	90.18	96.40	84.97	< 0.01
	KNN	85.39	73.68	71.22	85.39	85.39	98.25	74.63	< 0.01
	XGB	69.50	42.35	40.23	69.50	69.50	84.03	57.35	< 0.01
	Voting	87.01	76.13	74.31	87.01	87.01	97.80	77.98	< 0.01
	AMP-RNNpro	90.04	80.67	80.13	90.04	90.04	95.45	85.52	< 0.01
Pse-KRAAC	EX	82.13	65.54	64.53	82.13	82.13	90.04	75.51	< 0.01
	RF	**82.97**	66.19	65.94	82.97	82.97	86.09	80.37	< 0.01
	KNN	82.61	66.72	65.53	82.61	82.61	91.34	75.30	< 0.01
	XGB	64.33	34.68	30.97	64.33	64.33	86.39	45.88	< 0.01
	Voting	81.13	63.39	62.51	81.13	81.13	88.44	75.01	< 0.01
	AMP-RNNpro	75.27	54.08	51.50	75.27	75.27	89.94	63.00	< 0.01

Significant values are in bold.

In Table 2, from the various descriptors, it can be deduced that the best performance has been obtained from ASDC feature encoding, demonstrating as a potential candidate among the eight feature encoding techniques. From the AAC feature selection, the best outcome has been obtained by EX considering the overall evaluation metrics than the other models. AMP-RNNpro performed better than the other models, securing 95.58% accuracy, surpassing other models on ASDC features. It can be included that the AMP-RNNpro model performed remarkably not only with ASDC feature but also with additional features while considering all the evaluation metrics. In ASDC, the sensitivity and specificity of this model have been obtained respectively, 97.65% and 93.85%, which indicates proficiency in detecting a new sample precisely. Following that, in the CKSAAGP feature, AMP-RNNpro has performed considerably better than the other models, obtaining an accuracy of > 90%. In the DP feature encoding approach, EX has performed notably, providing an accuracy of 95.19% and the other evaluators scoring more than 90%. In GAAC encoding, AMP-RNNpro resulted in better performance than the other models. In the MORAN feature, both EX and AMP-RNNpro have performed well, resulting in a similar accuracy of 90.18%. But we calculated the other evaluation metrics where the EX model performed notably in consideration of the sensitivity and specificity, which is 96.45%, 84.93% on par with the AMP-RNNpro model, which has achieved 95.95% on sensitivity and 85.95% on specificity. In the NMBroto and PseKRAAC feature approaches, the RF model obtained the highest accuracy than the other models. It is prevalent that ASDC can provide enormous potential in detecting AMPS, whereas AMP-RNNpro displayed the most outstanding performance considering other classifires. Accordingly, all models are statistically significant, except for AMP-RNNpro of the DP descriptor, where the p-value is greater than 0.01, indicating that the model does not have sufficient methods to reject the null hypothesis. The study found that a p-value of less than 0.05 indicates scientific validation, which can result in a significant difference when making decisions^58^. All the p-values are included in the supplementary file.

In Table 3, we demonstrated the analysis of 48D probabilistic values, where we merged all the probabilistic values which are generated from our best six chosen model of machine learning method. However, this table showed that AMP-RNNpro has optimal performances than others, where AMP-RNNpro excels in performance and demonstrates excellent results in various evaluation metrics. This model exhibits accuracy in classifying tasks with a 97.15%. In K, a measurement of inter-rater consistency, indicates the model's stability with an exceptional value of 94.30%. The MCC of 94.31%. Furthermore, the model's capacity to accurately capture the positive class of 96.48% in Sn and specifically detect the negative class with 97.87% respectively. The model's balanced performance is indicated by the f1-score, precision, which achieves an astounding 97.23% with values of and 97.87%. Though in KNN and Voting has high precision rat but the AMP-RNNpro has optimal values in other assessments with adequate precision, where this model captured the actual class more than 97 times and balanced the actual class and the predicted class more precisely. In Sp, Voting has 98.34%, which is high performance to distinguish the negative classes from the samples, however, our proposed model AMP-RNNpro has potential performance to detect the non-AMPs. Overall, the AMP-RNNpro method is a suitable model for determination of antimicrobials from FASTA sequences.

**Table 3:** Performance analysis of probabilistic features frameworks.

Mode	Classifier	Accuracy	MCC	K	PR	FS	Sn	Sp
48D probabilistic features	EX	0.9703	0.9409	0.9407	0.9820	0.9711	0.9604	0.9810
	RF	0.9624	0.9248	0.9247	0.9709	0.9634	0.9560	0.9692
	KNN	0.9635	0.9278	0.9271	0.9839	0.9641	0.9450	0.9834
	XGB	0.9658	0.9316	0.9316	0.9732	0.9668	0.9604	0.9716
	Voting	0.9624	0.9257	0.9248	0.9839	0.9629	0.9428	0.9834
	**AMP-RNNpro**	**0.9715**	**0.9431**	**0.9430**	0.9799	**0.9723**	**0.9648**	0.9787

Significant values are in bold.

**Figure 4:**
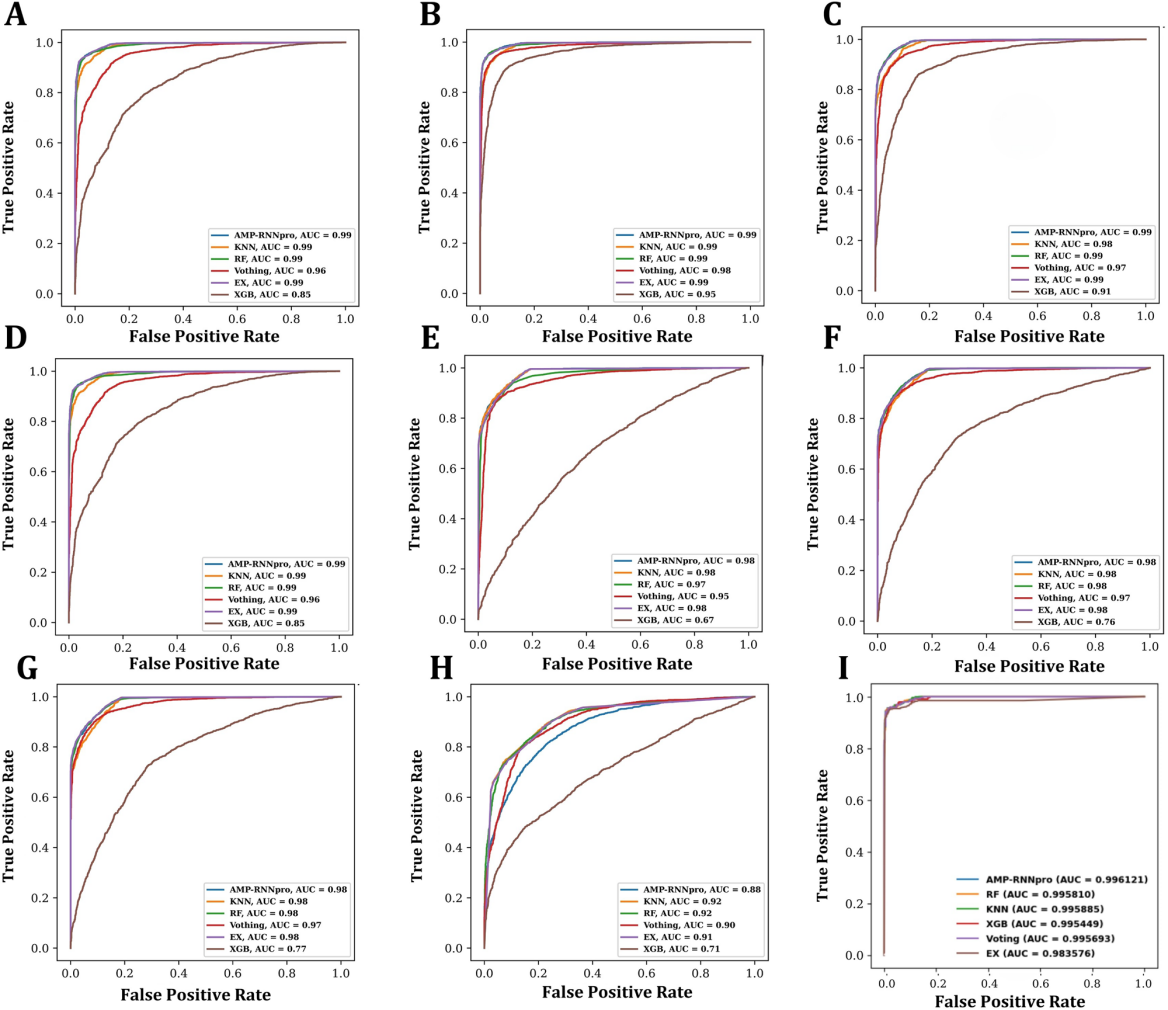
AUC Roc curve analysis on six classifiers on eight feature encoding methods and probabilistic merged dataset. The approaches are labeled **(A)** AAC feature **(B)** ASDC feature **(C)** CKSAAGP feature **(D)** DP feature **(E)** GAAC feature **(F)** MORAN feature **(G)** NMBroto feature **(H)** PseKRAAC feature **(I)** 48-dimensional probabilistic features from six classifier.

Figure 4 compares the true positive and true negative rates for six classifiers using eight feature encodings and probabilistic techniques (AAC, ASDC, CKSAAGP, DP, GAAC, MORAN, NMBroto, PseKRAAC, 48D probability merged dataset). The approaches are labeled A, B, C, D, E, F, G, H and I. When a thorough analysis is considered, AMP-RNNpro stands out as the best model inside the machine learning framework for feature encoding and 48D dataset. The RF, AMP-RNNpro, KNN, and Ex classifiers each attain a noteworthy AUC value of 0.99 in subplots A, B, and D. In C, the AMP-RNNpro, KNN, and Ex classifiers achieved 0.99 AUC score. The AMP-RNNpro, KNN, RF, and Ex classifiers have a remarkable AUC value of 0.98 in subplots of F, G. In the E subplot, The AMP-RNNpro, KNN, and Ex classifiers have 0.98 AUC value. KNN and RF classifiers have an AUC score of 0.92 in Subplot H. In I, we demonstrated the probabilistic values outcomes, where it is clearer that, with the probability values most of the models outperformed with this dataset, where AMP-RNNpro model obtained 99.61% of AUC score, demonstrates the proficiency in accurately distinguishing the classes. As a result, Fig. 4 illustrates the overall decent performance of these methods, with the majority identifying AMPs effectively with AUC values over 0.99.

### Comparison of AMP-RNNpro with others model in the current study

**Figure 5:**
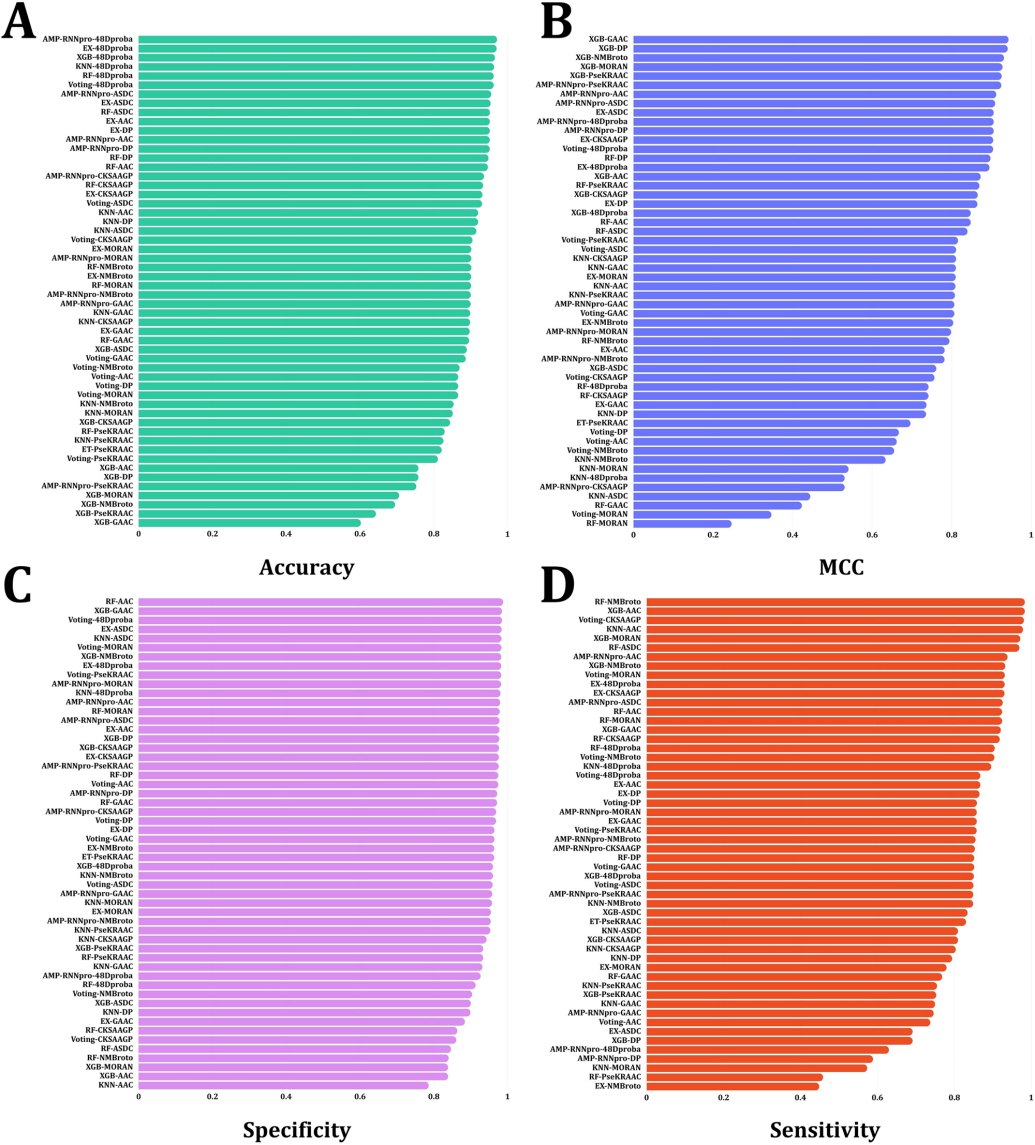
A comparison of 48D probability values classifieir with eight feature encodings classifier. The approaches are labeled as: **(A)** accuracy, **(B)** MCC, **(C)** specificity, **(D)** sensitivity.

To demonstrate the strengths of probabilistic feature combinations over single-feature encoding, we generated figures based on several performance evaluation metrics. Our study, represented in Fig. 5, arranges feature extraction strategies according to performance. It becomes prevalent for every performance evaluation metric that AMP-RNNpro outperforms every other single based model. Though in single-based descriptor’s XGB, and RF demonstrated an excellent performances in MCC, SP, and Sn, but overall performance consideration, we conclude that AMP-RNNpro model has optimal numbers with 48D probabilistic values but also this model well performed in single based feature encoding method, where, in accuracy term’s, AAC, ASDC, CKSAAGP has optimal performed with AMP-RNNpro, and with the probability this framework obtained higher accuracy than the others method. In MCC, SN, and Sp have also this model provided a sufficient results. Therefore, considering the overall performances, we conclude that our proposed model AMP-RNNpro achieved a better outcome in every evaluation metrics with an adequate performance.

## Discussion

### Performance comparison of the existing predictor

**Figure 6:**
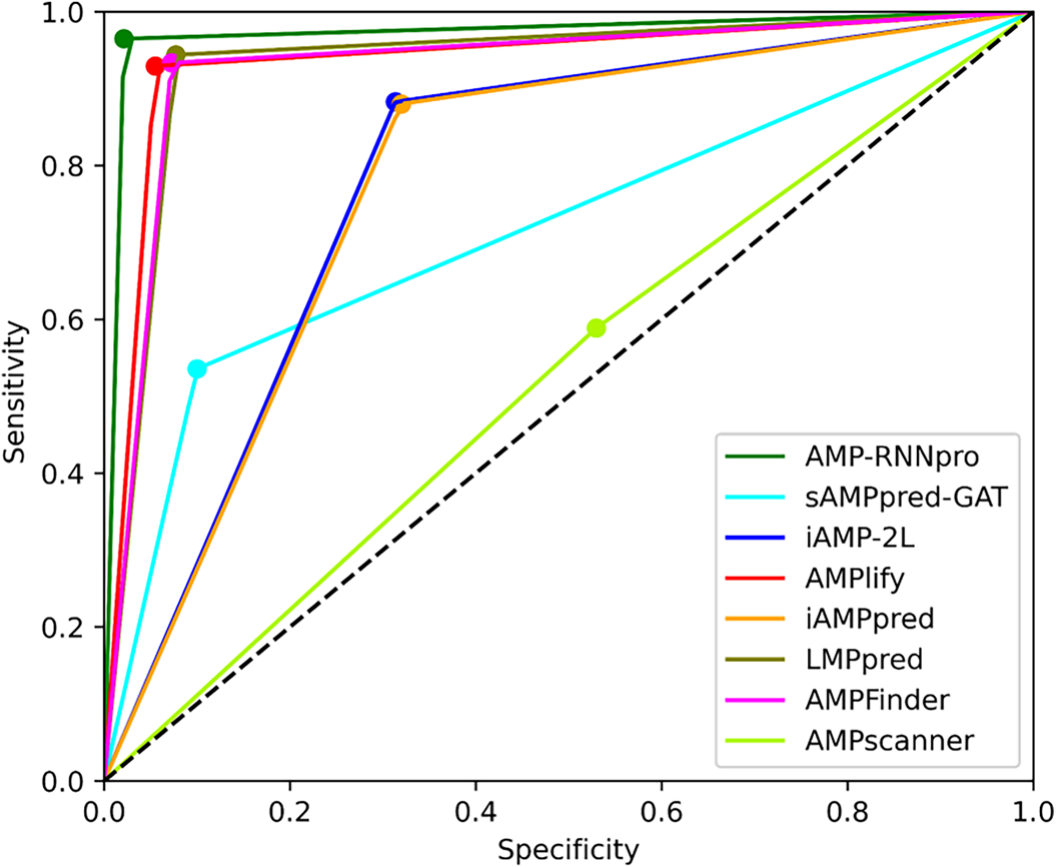
Comparison of the other proposed model with AMP-RNNpro.

Figure 6 illustrates a comprehensive comparison of specificity and sensitivity outcomes in several models including our proposed model and other existing models such as sAMPpred-GAT, iAMP-2L, AMPlify, iAMPpred, LMpred, AMPFinder, AMPscanner. The results show that our model, AMP-RNNpro, outperformed all other models. The increased specificity indicates that our algorithm correctly detects AMPs.

In Table 4, we have shown performance comparisons of our model with several existing prediction tools. It demonstrates that our model achieved higher accuracy and AUC scores than the other proposed models. Our proposed model has taken probabilistic features derived from 8 feature encoding techniques which possess intrinsic differentiating capability and delivered a composed outcome by identifying the negative class with 97.87% specificity and the positive class with 96.48% sensitivity. Moreover, our model has obtained a 99.61% AUC score and 97.15% accuracy. So, it can be concluded that our model has optimally distinguished between the active and inactive AMPs. In comparison with the iAMPred and iAMP-2L models’ performance on the independent test dataset of AMPs, our model has an increase in accuracy by 4% and specificity of 10% over these two models. Based on the independent test analysis, AMP-RNNpro outperformed AMPlify model by 15% in accuracy and 30% in sensitivity. The difference between sensitivity and specificity of AMPlify is over 30 percent that may lead to an unbalanced detection on unseen data. Our suggested model is more powerful and more accessible to detect the AMPs than the complex GAT-based feature selections model sAMPpred-GAT which used cross-validation method for evaluation. In our study, we have evaluated our model based on independent test as it is more viable to depict how suited our model is for practical application than the cross-validation technique. However, sAMPpred-GAT model's performances are relatively lower than AMP-RNNpro and also possess difference between sensitivity and specificity over 35% that may greatly affect to the unbiasedness of the model. LMpred and AMPfinder tested their models on various datasets. AMP-RNNpro outperformed LMpred by 3 percent in accuracy, sensitivity, and specificity. In comparison with AMPfinder, AMP-RNNpro achieved 3% higher results in accuracy. In AMPfinder model’s performance, the gap between specificity and sensitivity is 10% whereas in our model it is 1% which demonstrates a more consistent performance in differentiating between the AMPs and non-AMPs. By comparing our proposed model to the majority of the state-of-the-art, we can conclude that our proposed model can successfully deliver more balanced and accurate results which will be more efficient for real life applications.

**Table 4:** Performances of AMP-RNNpro and existing AMPs prediction tools.

Model name	Accuracy	Sensitivity	Specificity	AUC	Reference
iAMPpred	0.9217	0.9938	0.8456	0.9361	^14^
iAMP-2L	0.9282	0.9956	0.8608	0.9018	^59^
AMPlify	0.8032	0.6162	0.9902	97.44	^19^
sAMPpred-GAT	0.715 $\pm$ 0.01	0.530 $\pm$ 0.011	0.9 $\pm$ 0.02	0.77	^21^
AMPFinder	0.9445	0.9945	0.8945	0.9874	^26^
LMPred	0.9333	0.9228	0.9438	0.9789	^20^
AMPscanner	0.5296	0.5885	0.4707	0.5436	^15^
AMP-RNNpro	0.9715	0.9648	0.9787	0.9961	Proposed model

### Adaptability and stability analysis

We conducted experiments with our proposed model on a diverse dataset. We experimented with AMPFinder's D1 test dataset and iAMPCN's initial stages test dataset to evaluate the model's capabilities with these datasets.

#### Case study 1

We used AMPFinders D1’s dataset^26^, and we observed that there were 980 active sequences and 982 non-active sequences. To validate our model with the dataset, we have recognized that AMP-RNNpro obtained 96.73% in accuracy, 99.82% in sensitivity, and 62.96% in specificity. It is clearly observed that our model performed well in the independent test approach.

#### Case study 2

We have another experiment with the iAMPCN^22^ models on a first-stage independent test dataset to validate our models. The authors stated that they organized their dataset by aggregating the various data repositories. However, we collected 2000 negative and positive samples to assess our model. The results of this study showed 96.13% in accuracy, 91.16% in sensitivity, and 98.46% in specificity. This result demonstrated our model's remarkable and potent ability to recognize the AMPs dataset.

### Interpretation

**Figure 7:**
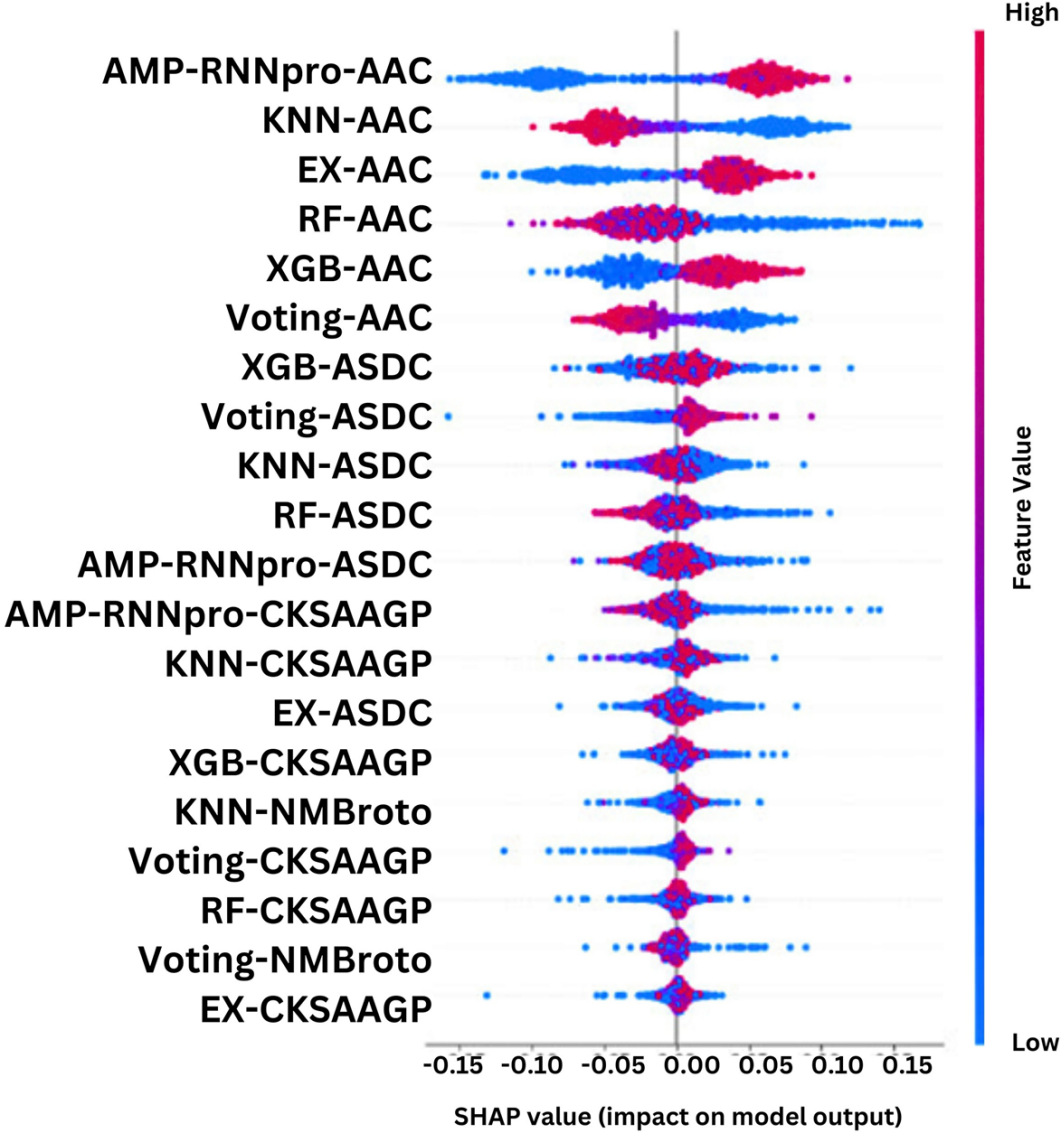
Features importance on top 20 features.

AMP-RNNpro has been constructed with optimal probabilistic features from eight feature encoding techniques. Hence, it has delivered a more robust and precise performance compared to the previous predictors. Following recent studies, a model interpretation by illustrating the impacts of the probabilistic features on performance has been accomplished using SHAP^30^. In Fig. 7, the illustration demonstrates the top 20 features based on their overall impact on the outcome of our model.

In Fig. 7, best six models based on AAC and ASDC, CKSAAGP features indicate their most significant contribution in the detection of AMPs. The remaining 2 spots of the top 20 have been taken by models based on the NMBroto feature encoding technique. So, it dictates that the compositional features of AAC and ASDC play a vital role in the detection and development of medications. Wang et al. previously conducted AAC, the amino acid composition and ASDC which represents the amino acid chain. The authors stated that these two features have significant potential for drug discoveries and peptide identification^60^. Kabir et al. also mentioned that the AAC feature is more impactful in detecting AMPs^61^. Park et al. proposed an antimicrobial function: anticancer prediction tools, The study found that CKSAAGP was one of the most important features for predicting the anticancer^62^. As a result, it can be apprehended that the further exploration of these features holds greater possibilities both in detection and drug discovery.

### Website implementation

**Figure 8:**
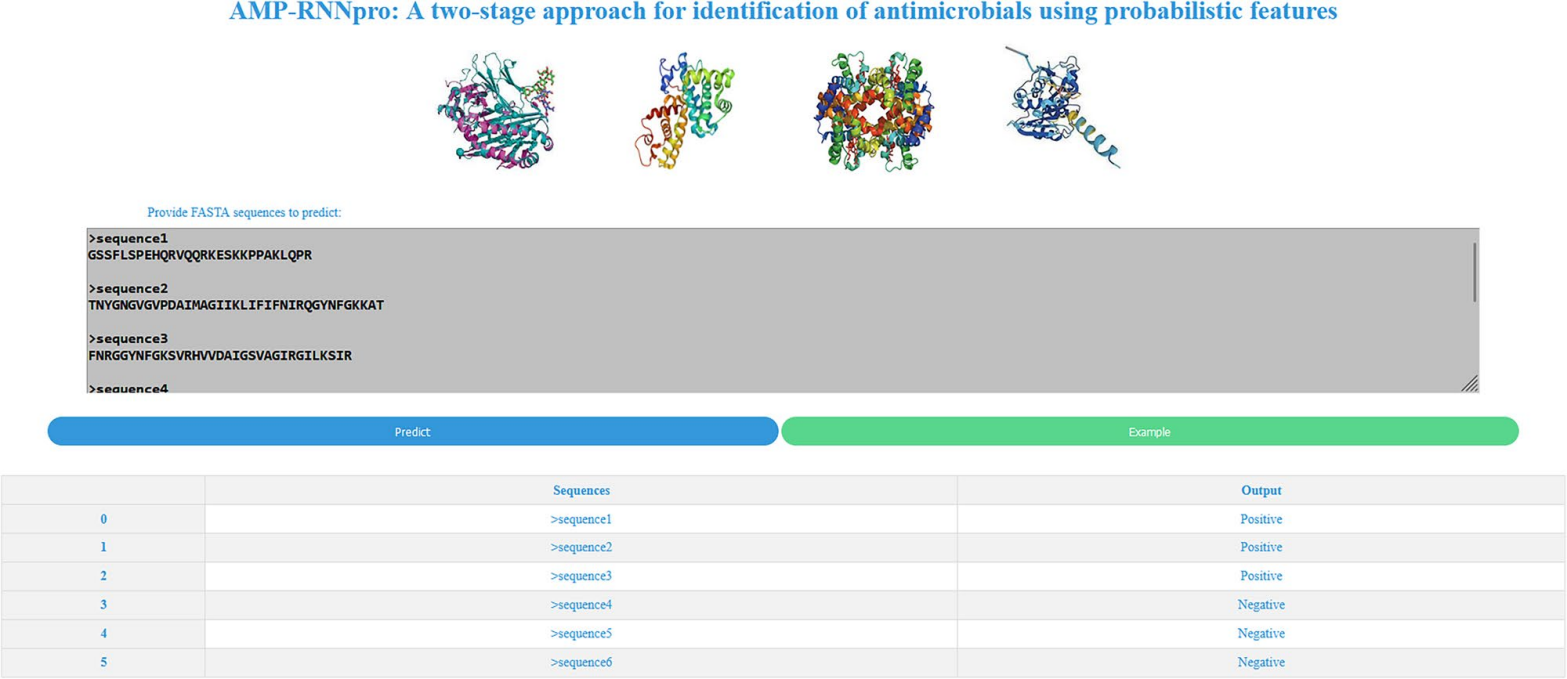
AMP-RNNpro framework’s website. Demonstrates a input box, example button, predict button and outcome of the AMPs.

We have implemented a website of our model to predict the AMPs. The interface of our prediction tool is shown in Fig. 8.

We have designed a simple interface that is easier to understand and efficient to use for detecting AMPs with proper functionalities. Initially, an input section is given, allowing a user to provide sequences in FASTA format for AMP prediction of the AMPs. Below the input section are two buttons: ‘Predict’ and ‘Example’. After clicking the ‘Predict’ button, it shows the prediction result in the output box. The output is shown in the following First-In-First-Out (FIFO) format. When the user presses ‘Example’ button it will give some sequences in the input section. The output will be shown as positive for the active AMPs and negative for the inactive-AMPs. Additionally, if the given sequences contain any unnecessary numbers or strings then the excessive numbers or strings will be excluded while the prediction and the result will be provided for the clipped sequences. Our prediction tool can be found at http://13.126.159.30/.

## Conclusion

A robust and novel method, named AMP-RNNpro, has been developed for detecting AMPs based on eight features of different criteria, additionally providing insights into the features that play a dominant role in the detection. The proposed model comprises compositional, positional, and physiochemical, as well as other properties for detecting AMPs with high accuracy and precision. Our recommended method is novel as the probabilistic features possess more innate abilities to distinguish AMPs. Thus, it analyzes AMPs more swiftly, instantly identifying if they have anti-characteristics and categorizing the features. In healthcare institutions, it is crucial for efficiently and rapidly appraising patient medication. We have built a user-friendly website to predict the AMPs with our proposed model.

To increase the precision and efficiency of AMP identification, future studies are needed to explore new feature encoding methods and ensembled deep neural networks feature selection techniques that may help in measuring the contribution of each feature encoding technique in discerning AMPs from non-AMPS considering the incorporation of larger datasets from the medical field.

### Supplementary Information

Supplementary Information 1.

Supplementary Information 2.

## Data Availability

The dataset and the source code have been available for this study is here. https://github.com/Shazzad-Shaon3404/Antimicrobials_.git.

## Abbreviations

AMPs: Antimicrobial peptides
RNN: Recurrent neural network
KNN: K-nearest neighbor
RF: Random forest
XGB: Extreme gradient boosting classifier
EX: Extra-tress classifier
CD-HIT: Cluster database at high identity with tolerance
AAC: Amino acid composition
ASDC: Adaptive skip dinucleotide composition
GAAC: Grouped amino acid composition
CKSAAGP: Composition of K-spaced amino acid pairs
DP: PseAAC of distance-pairs and reduced alphabet
PseKRAAC: Pseudo K-tuple reduced amino acid composition
MORAN: Moran autocorrelation
NMBroto: Normalized Moreau–Broto
MCC: Matthews correlation coefficient
Sn: Sensitivity
Sp: Specificity
K: Kappa score
FS: F1 score
PR: Precision
RE: Recall
AUC: Area under the curve
