# Supplementary material for: AMP-RNNpro: a two-stage approach for identification of antimicrobials using probabilistic features
Source: Sci Rep. 2024 Jun 5;14:12892. doi: 10.1038/s41598-024-63461-6 (PMC11153637; doi:10.1038/s41598-024-63461-6)
Supplement: Supplementary file 2 — Supplementary Information 2. [file 41598_2024_63461_MOESM2_ESM.zip › Supplementary file 2.docx]

**P-values of the algorithms**

| **Descriptor** | **Classifier** | **P values** | | | | | | | |
| --- | --- | --- | --- | --- | --- | --- | --- | --- | --- |
| AAC | EX | pvalue=0.0005528543098201366 | | | | | | | |
|  | RF | pvalue=1.8423475013773968e-07 | | | | | | | |
|  | KNN | pvalue=5.412253240321548e-07 | | | | | | | |
|  | XGB | pvalue=4.023251030484647e-05 | | | | | | | |
|  | Voting | pvalue=6.929746219592158e-17 | | | | | | | |
|  | StackMP | pvalue=0.008540700054533123 | | | | | | | |

| **Descriptor** | **Classifier** | **P values** | | | | | | | |
| --- | --- | --- | --- | --- | --- | --- | --- | --- | --- |
| ASDC | EX | pvalue=0.0014475175291771586 | | | | | | | |
|  | RF | pvalue=3.881604106297472e-06 | | | | | | | |
|  | KNN | pvalue=2.746444369603812e-08 | | | | | | | |
|  | XGB | pvalue=0.005065589362341345 | | | | | | | |
|  | Voting | pvalue=2.0292312609641255e-15 | | | | | | | |
|  | StackMP | pvalue=0.000335168013565518 | | | | | | | |

| **Descriptor** | **Classifier** | **P values** | | | | | | | |
| --- | --- | --- | --- | --- | --- | --- | --- | --- | --- |
| CKSAAGP | EX | pvalue=0.00015269566322616423 | | | | | | | |
|  | RF | pvalue=1.5888947899165767e-09 | | | | | | | |
|  | KNN | pvalue=8.305764372355167e-09 | | | | | | | |
|  | XGB | pvalue=0.0004414764884690336 | | | | | | | |
|  | Voting | pvalue=1.3942007308012305e-19 | | | | | | | |
|  | StackMP | pvalue=1.0003716791604274628e-14 | | | | | | | |

| **Descriptor** | **Classifier** | **P values** | | | | | | | |
| --- | --- | --- | --- | --- | --- | --- | --- | --- | --- |
| DP | EX | pvalue=0.0005528543098201366 | | | | | | | |
|  | RF | pvalue=4.2586468572988043e-07 | | | | | | | |
|  | KNN | pvalue=5.418005942645554e-07 | | | | | | | |
|  | XGB | pvalue=4.023251030484647e-05 | | | | | | | |
|  | Voting | pvalue=6.933186072646605e-17 | | | | | | | |
|  | **StackMP** | **pvalue=0.071558727510977** | | | | | | | |

| **Descriptor** | **Classifier** | **P values** | | | | | | | |
| --- | --- | --- | --- | --- | --- | --- | --- | --- | --- |
| GAAC | EX | pvalue=1.6306205683764223e-05 | | | | | | | |
|  | RF | pvalue=2.2864734660537147e-10 | | | | | | | |
|  | KNN | pvalue=3.2112835072554428e-06 | | | | | | | |
|  | XGB | pvalue=2.9479359067147257e-13 | | | | | | | |
|  | Voting | pvalue=2.3768026821485196e-20 | | | | | | | |
|  | StackMP | pvalue=1.8572393199943125e-09 | | | | | | | |

| **Descriptor** | **Classifier** | **P values** | | | | | | | |
| --- | --- | --- | --- | --- | --- | --- | --- | --- | --- |
| MORAN | EX | pvalue=2.6819988839132868e-06 | | | | | | | |
|  | RF | pvalue=2.9977397380256976e-11 | | | | | | | |
|  | KNN | pvalue=5.435631193175066e-12 | | | | | | | |
|  | XGB | pvalue=9.526236029996758e-11 | | | | | | | |
|  | Voting | pvalue=4.788361321465521e-25 | | | | | | | |
|  | StackMP | pvalue=1.1561649090412692e-05 | | | | | | | |

| **Descriptor** | **Classifier** | **P values** | | | | | | | |
| --- | --- | --- | --- | --- | --- | --- | --- | --- | --- |
| NMBroto | EX | pvalue=1.9121168630869354e-06 | | | | | | | |
|  | RF | pvalue=8.391100247781762e-12 | | | | | | | |
|  | KNN | pvalue=1.1695650977930215e-10 | | | | | | | |
|  | XGB | pvalue=1.1378323173948503e-11 | | | | | | | |
|  | Voting | pvalue=1.452925910375542e-23 | | | | | | | |
|  | StackMP | pvalue=1.2417320994671848e-06 | | | | | | | |

| **Descriptor** | **Classifier** | **P values** | | | | | | | |
| --- | --- | --- | --- | --- | --- | --- | --- | --- | --- |
| PseKRAAC | EX | pvalue=0.0009608329814956675 | | | | | | | |
|  | RF | pvalue=1.3774713947427154e-05 | | | | | | | |
|  | KNN | pvalue=0.0009269154942948613 | | | | | | | |
|  | XGB | pvalue=2.265226726254519e-08 | | | | | | | |
|  | Voting | pvalue=3.860114178562876e-12 | | | | | | | |
|  | StackMP | pvalue=5.025362904315337e-18 | | | | | | | |
